# Comparison of the effects of metformin, citral, *Cymbopogon citratus* extract and silver nanoparticles of *Cymbopogon citratus* extract on oxidative stress indices and Nrf2 levels in experimental type 2 diabetes in rats

**DOI:** 10.1002/ame2.70017

**Published:** 2025-05-05

**Authors:** Milad Faraji, Mohammad Foad Noorbakhsh, Nasrin Kazemipour, Saeed Nazifi, Hamid Reza Moradi, Nasrollah Ahmadi, Maryam Azadmanesh

**Affiliations:** ^1^ Department of Basic Sciences, School of Veterinary Medicine Shiraz University Shiraz Iran; ^2^ Department of Clinical Sciences, School of Veterinary Medicine Shiraz University Shiraz Iran; ^3^ Department of Pathobiology, School of Veterinary Medicine Shiraz University Shiraz Iran

**Keywords:** diabetes, lemongrass, metformin, Nano particle, Nrf2, oxidative stress

## Abstract

**Background:**

Diabetes, a metabolic disease marked by endocrine dysfunctions, significantly impacts oxidative stress pathways. This study explores the protective effects of lemongrass extract (LE), citral, and lemongrass extract‐synthesized silver nanoparticles (LE‐AgNP) against hyperglycemia‐induced oxidative stress by modulating the Nrf2 pathway, which is crucial for antioxidant responses.

**Methods:**

Various techniques characterized the nanoparticles, including ultraviolet–visible spectroscopy, dynamic light scattering, zeta potential analysis, Fourier‐transform infrared spectroscopy, scanning electron microscopy and transmission electron microscopy. Seventy rats, divided into seven groups, were used for in vivo experiments. Type 2 diabetes was induced using streptozotocin (65 mg/kg) and nicotinamide (90 mg/kg). After six weeks, biochemical parameters such as total antioxidant capacity, malondialdehyde, Nrf2, and Nrf2 miRNA were evaluated in pancreas tissue and blood serum, along with serum blood glucose levels.

**Results:**

LE‐AgNP significantly improved weight gain, reduced food and water intake, and lowered blood glucose levels in diabetic rats. LE and citral did not significantly alter these parameters but prevented the decline in Nrf2 gene expression. LE‐AgNP showed a significant increase in Nrf2 gene expression compared to the diabetic control group.

**Conclusions:**

This study highlights the potential of LE, citral, and especially LE‐AgNP in mitigating oxidative stress induced by diabetes. LE‐AgNP demonstrated superior therapeutic benefits, including improved oxidative stress conditions and hypoglycemic effects.

## Graphical abstract

The experimental type 2 diabetes model in rats induced by streptozotocin and nicotinamide showed that silver nanoparticles synthesized from lemongrass extract modulate blood glucose, water and food intake, and enhance antioxidant enzyme synthesis and weight gains.

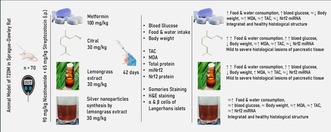

## 1 INTRODUCTION

Diabetes is rapidly becoming one of the most prevalent diseases globally, with projections indicating it will impact 693 million adults by 2045.^1^ Type 2 diabetes, the most common form, is characterized by chronic hyperglycemia due to impaired glucose metabolism and defects in insulin secretion and activity.^2^ Pancreatic cells have low antioxidant enzyme levels, making them vulnerable to oxidative stress.^3^ Elevated blood glucose levels in diabetes increase the production of reactive oxygen species (ROS), leading to oxidative damage.^4^

The nuclear factor (erythroid‐derived 2)‐like 2 (Nrf2) is crucial in diabetes management and acts by activating antioxidant defense systems through the transcription of various antioxidant genes, reducing oxidative stress and benefiting both microvascular and macrovascular complications.^5^ However, Nrf2 levels and activity can vary depending on diabetes conditions, including the timing and duration of activation, and whether the condition is acute or chronic.^6^ Dysfunction or absence of Nrf2 signaling worsens both type 1 and type 2 diabetes, underscoring its potential as a therapeutic target.^6^, ^7^

Recent research highlights plant compounds with hypoglycemic and hypolipidemic effects for type 2 diabetes treatment. These compounds reduce fat, lower blood glucose, and have antioxidant properties.^8^ *Cymbopogon citratus* (lemongrass) is known for its antioxidant, anti‐diabetic, and anti‐inflammatory properties.^9^ Studies show that aqueous extracts of lemongrass leaves have hypoglycemic and hypolipidemic effects in diabetic rats, suggesting their potential use for type 2 diabetes.^10^ Citral, the key component in lemongrass oil, has notable anti‐inflammatory and antioxidant effects, lowering lipid oxidation and ROS levels while boosting antioxidant capacity. These qualities make it a promising supplementary treatment for diabetes.^11^, ^12^ Studies show lemongrass extract scavenges free radicals, and citral administration in diabetic rats significantly reduces blood glucose, enhances insulin levels, and improves oxidative markers.^13^, ^14^ To enhance the stability and efficacy of plant compounds, nanoparticle synthesis has emerged as a solution. Nanoparticles increase the solubility of hydrophobic drugs, enhance loading capacity, and improve bioavailability, thereby maintaining the drug's effectiveness and stability.^15^, ^16^ Green synthesis of nanoparticles using plant extracts offers an environmentally friendly alternative to chemical methods, reducing pollution and enhancing safety.^17^ Silver nanoparticles synthesized from lemongrass extract have demonstrated anti‐diabetic properties, making them a promising area of study.^18^

Experimental models, such as streptozotocin/nicotinamide‐induced diabetes in rats, are widely used to mimic human type 2 diabetes by partially damaging pancreatic β‐cells, leading to hyperglycemia and insulin resistance. These models provide a reliable platform for studying disease mechanisms and testing potential therapeutic agents.^19^

This study compares the effects of lemongrass extracts, lemongrass‐synthesized silver nanoparticles, and citral in a rat model of streptozotocin/nicotinamide‐induced diabetes.

## 2 METHODS

### 2.1 Chemicals and reagents

Nicotinamide (Lot. No.: BCCD3340) and streptozotocin (Lot. No.: WXBD4971V) were from Sigma Chemical Co.. Citral (Cas. No.:5392‐40‐5), metformin (Lot. No.: W1117214), silver nitrate (Cas. No.:7761‐88‐8) and methanol (Cas. No.:67–56‐1) were obtained from Merck.

### 2.2 Extracting

Lemongrass was collected from Fars province, Iran, and verified by the Agricultural Faculty of Shiraz University. After washing the leaves with distilled water, they were air‐dried in a dark environment. The dried leaves were cut into smaller pieces, mixed with distilled water in a 1:8 ratio, and boiled for 30 minutes. The solution was then filtered using Whatman No. 1 and freeze‐dried to obtain a powder.^18^, ^20^

### 2.3 Eco‐friendly production of silver nanoparticles using lemongrass extract (LE‐AgNP)

For the production of these particles, a bio‐regeneration approach, which relies on chemical and biological methods, was used.^21^ For the fabrication of silver nanoparticles through lemongrass extract (LE), we first prepared a 1 mmol/L solution of silver nitrate (AgNO_3_) in deionized water. This was then mixed with prepared LE (10 mg/mL) at ratios of 1:99, 2:98, 1:199 and 1:499 and the resulting solutions were placed on a stirrer for 24 h.

### 2.4 Experimental animals

The study involved 70 healthy male Sprague–Dawley rats, obtained from the Laboratory Animal Center of Shiraz University of Medical Sciences. The rats were 1.5 months old, with an average weight of 250 ± 50 g. They were housed in a controlled environment with a 12‐h light/dark cycle at a temperature of 23 ± 1°C. Two rats were kept per cage, with ad libitum access to standard food (Behparvar Industrial Expansion & Development Co) and water. To ensure acclimatization, the animals were housed in the facility for one week prior to the initiation of the experiment.

### 2.5 Experimental diabetes induction

To induce type 2 diabetes mellitus (T2DM) in rats, after a 12‐hour fasting period, a single intraperitoneal (i.p.) injection of streptozotocin (STZ) at 65 mg/kg body weight, dissolved in cold citrate buffer (100 mmol/L, pH 4.5), was administered. This was done 15 minutes after an intraperitoneal injection of nicotinamide (NA) at 90 mg/kg body weight.^22^ To prevent fatal hypoglycemia from increased pancreatic insulin secretion induced by STZ, rats received a 5% glucose solution for 24 h, starting 6 h after the STZ injection. Blood glucose levels were measured three days later, and rats with levels above 250 mg/dL were classified as diabetic.

### 2.6 Study methodology

The rats were randomly divided into seven groups, each consisting of ten rats:

- Control: The rats received an intraperitoneal injection of 0.5 mL normal saline on the first day, followed by 1 mL of normal saline via oral gavage daily to simulate the same injection and gavage stress as the other groups.
- Cosolvent Control: The rats initially received STZ and NA. The diabetic rats were then given 1 mL of citral carrier (olive oil) via oral gavage daily for the duration of the experiment.
- T2DM: The diabetic control group received STZ and NA but no further treatment.
- Metformin: Diabetic rats were treated with 100 mg/kg body weight of metformin in an aqueous suspension via oral gavage daily for 6 weeks.
- LE: Diabetic rats were given 30 mg/kg body weight of lemongrass extract in an aqueous suspension via oral gavage daily for 6 weeks.
- Citral: Diabetic rats were treated with 30 mg/kg body weight of citral in an oil suspension via oral gavage daily for 6 weeks.
- LE‐AgNP: Diabetic rats received 30 mg/kg body weight of lemongrass extract conjugated with silver nanoparticles in an aqueous suspension via oral gavage daily for 6 weeks.

During the study, key parameters like body weight, blood glucose, and intake levels were monitored. Dosages were adjusted weekly based on changes in body weight. After the 6 weeks, rats were euthanized (ketamine 80 mg/kg, xylazine 10 mg/kg) for blood collection and pancreatic tissue extraction. The pancreas was stored at −80°C, with part of the tissue preserved in 10% buffered formalin at room temperature for histological examination.

### 2.7 Preparation of pancreatic tissue homogenate

The pancreatic tissue was homogenized using liquid nitrogen. The tissue was weighed (100 mg), frozen with liquid nitrogen, and crushed using a mortar. The crushed tissue was then mixed with phosphate‐buffered saline (1 mol/L, pH 7.4) at a ratio of 1:10. The mixture was centrifuged at 4032*g* for 10 min at 6°C, after which the supernatant was collected for further analysis.

### 2.8 Biochemical assays of serum and tissue

Serum glucose (Lot. No.: 1400–12) and total protein (Lot. No.: 1400–10) levels were measured using commercial kits (Pars Azmun Co.) and a biochemical auto‐analyzer (Alpha Classic AT++). Nrf2 (Lot. No.: ZB‐OER245211103‐121), total antioxidant capacity (TAC, Lot. No.: ZB‐A1211019), and malondialdehyde (MDA, Lot. No.: ZB‐A3220404) levels in serum were determined using commercial kits (Zelbio Co.) following the manufacturer's protocols. For tissue analysis, total protein concentration in the pancreas was quantified by the biuret technique,^23^ and Nrf2, MDA, and TAC levels were measured using Zelbio kits. Both serum and tissue results were evaluated with an ELISA microplate reader (BioTek Power Wave XS2), and the concentrations were calculated accordingly.

### 2.9 Histological studies

Routine paraffin wax sectioning and Hematoxylin–Eosin (H&E) and Gomori staining methods were used for histological and pathological analysis.^24^ Tissue sections were cut to a thickness of 4–6 μm. Some sections were stained with H&E for general pathological examination, while others were stained with aldehyde‐fuchsin to observe beta and alpha cells in the islets of Langerhans. All slides were examined under a bright field microscope (Olympus CX21), and images were captured using a dedicated microscope camera (Olympus E450).

### 2.10 RNA isolation and quantitative real‐time PCR

Total RNA from the pancreas tissues (30 mg) was extracted using the FavorPrep™ Tissue Total RNA Mini Kit. The purity was ensured to be within the ratio of 1.8–2.0 at A260/A280. Then, the total RNA was reverse transcribed into cDNA using the RevertAid First Strand cDNA Synthesis Kit (Thermo Scientific, Waltham, MA, USA) and run into a StepOnePlus™ Real‐Time PCR System (Applied Biosystems™). Primers were designed using Allele IDv7.8 software, with the following sequences:

- Nrf2 (Accession Numbers: NM_031789.3): forward 5′‐CACATCCAGACAGACACCAGTC‐3′ and reverse 5′‐CTACAAATGGGAATGTCTCTGC‐3′
- TATA‐binding protein (Accession Numbers: NM_001004198.1): forward 5′‐GCGGGGTCATGAAATCCAGT‐3′ and reverse 5′‐AGTGATGTGGGGACAAAACGA‐3′, used as an internal control.

The relative expression level of the Nrf2 gene compared to TATA‐binding protein (TBP) and the control group was determined using the ΔΔCt method. The gene expression was quantified using the formula 2^−ΔΔCt^.

### 2.11 Statistical analysis

The normality of the data was assessed using the Kolmogorov–Smirnov test. The results were expressed as mean ± SEM, and statistical significance was evaluated using ANOVA with the GraphPad Prism program (version 10.1). Post hoc analysis was conducted using the Tukey's test. Statistical significance was defined as *p* < 0.05.

## 3 RESULTS

### 3.1 Evaluation and characterization of silver nanoparticles

**FIGURE 1:**
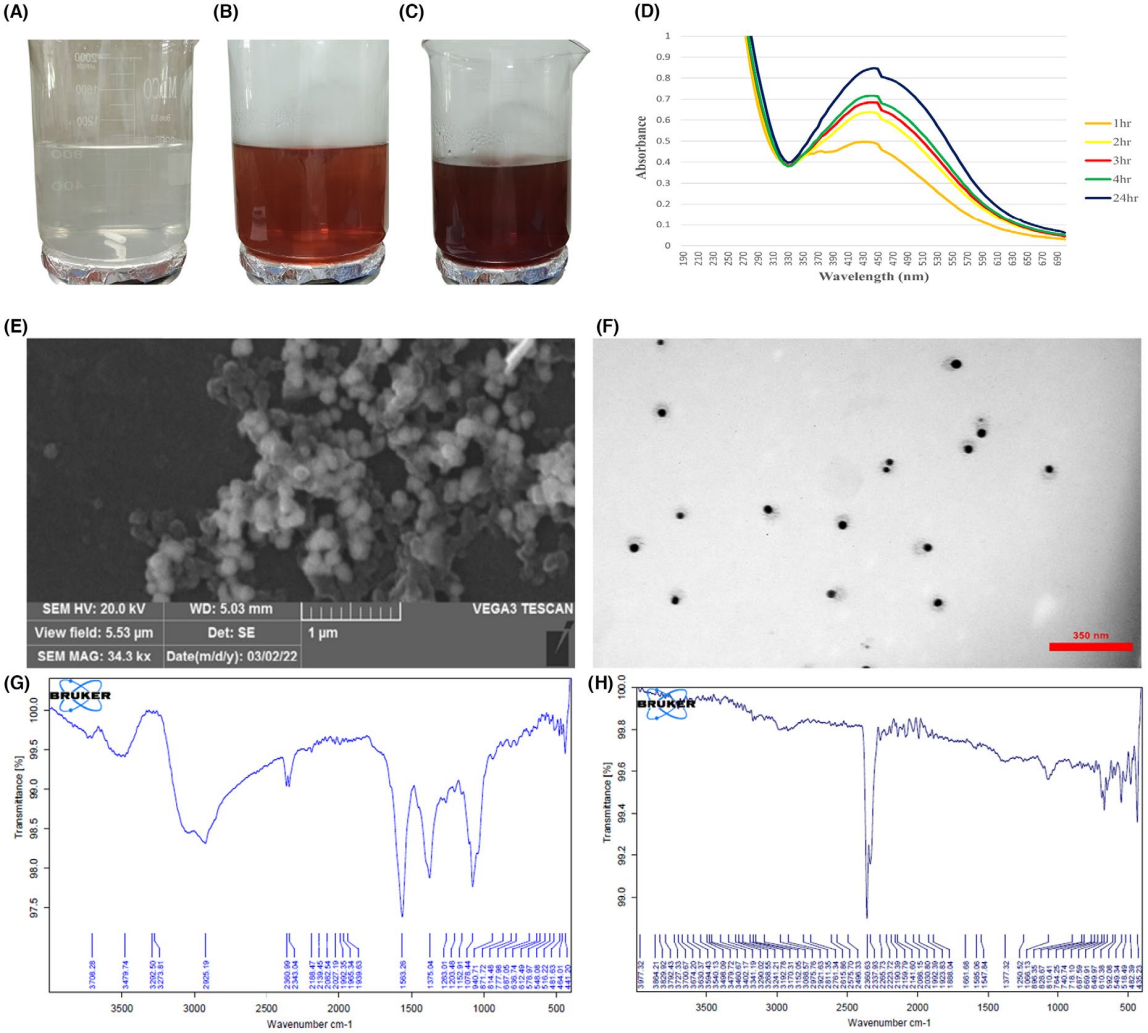
Evaluation and characterization of silver nanoparticles synthesized using cymbopogon citratus extract. (A) The color change of a 1 mmol\L silver nitrate solution mixed with a solution containing 10 mg/mL lemongrass extract (ratio 1:499). (B) Color change after 1 h of stirring. (C) Color change after 4 h of stirring. (D) UV–visible spectrophotometric analysis, with a ratio of 1:499 observed after 4 h of stirring. (E) Scanning electron microscopy image. (F) Transmission electron microscopy image. Magnification: 27800x. (G) Fourier‐transform infrared (FTIR) spectra of fresh cymbopogon citratus extract. (H) FTIR spectra of silver nanoparticles synthesized using the cymbopogon citratus extract. [Correction added on 04 Sep 2025 after first online publication: The magnification level has been added in the figure 1F caption and figure 4 is updated.]

The change in the color of the silver nitrate solution‐LE mixture to light brown (Figure 1A–C) indicated the formation of nanoparticles. This was further confirmed by measuring the optical absorption of the solution using UV–Vis spectroscopy over several hours within the 200–700 nm wavelength range. An absorption peak at 440 nm (Figure 1D) was observed, consistent with the formation of silver nanoparticles according to Mie scattering theory.^25^

Dynamic light scattering (DLS) measurements revealed that the average sizes (mean ± SD) of the synthesized silver nanoparticles for the mixture ratios of 1:99, 2:98, 1:199, and 1:499 were 289.6 ± 132, 165.8 ± 12.8, 104.1 ± 21.9, and 68.5 ± 17.2 nm, respectively. The dispersion medium's viscosity was 0.894 mPa·s, and the zeta potential for the 1:499 ratio was −30.4 mV. Based on DLS and UV–Vis spectra results, the 1:499 ratio was selected for fabricating silver nanoparticles using LE.

Scanning electron microscopy examination is a useful and powerful tool for identifying the morphology of nanoparticles. As shown in Figure 1E, the structural characteristics of silver nanoparticles synthesized from LE is almost spherical and irregular in shape.

The transmission electron microscope image in Figure 1F clearly shows the spherical structure with a diameter range of 36.24 ± 6.59 (mean ± SD) and the polydisperse nature of the silver nanoparticles synthesized from LE.

Fourier transform infrared (FT‐IR) measurements were performed to investigate the main functional groups in LE and LE‐AgNP. The FT‐IR spectrum of LE (Figure 1G) displayed strong bond stretching at various peaks, including 3479.74 cm^−1^ (N–H stretching), 2925.19 cm^−1^ (C–H), 2343.04 cm^−1^ (O=C=O), 1563.26 cm^−1^ (N–O), 1375.04 cm^−1^ (O–H), and 1078.44 cm^−1^ (C=O). In contrast, the FT‐IR spectrum of LE‐AgNP (Figure 1H) showed lower absorption values due to the low extract concentration used in nanoparticle synthesis, resulting in fewer functional groups. The peak at 1077.44 cm^−1^, corresponding to C–N stretching in LE, shifted to 1066.13 cm^−1^ in LE‐AgNP, suggesting that the amino group is crucial in the reduction process during nanoparticle synthesis. LE‐AgNP also exhibited notable peaks at 2975.76 cm^−1^ (C–H stretching), 1661.68 cm^−1^ (C=O stretching of NH), and 1377.32 cm^−1^ (C–C and C–N stretching), indicating that functional groups in LE acted as both reducing and stabilizing agents for the silver nanoparticles.

### 3.2 Changes in body weight, blood glucose, food, and water intake

**FIGURE 2:**
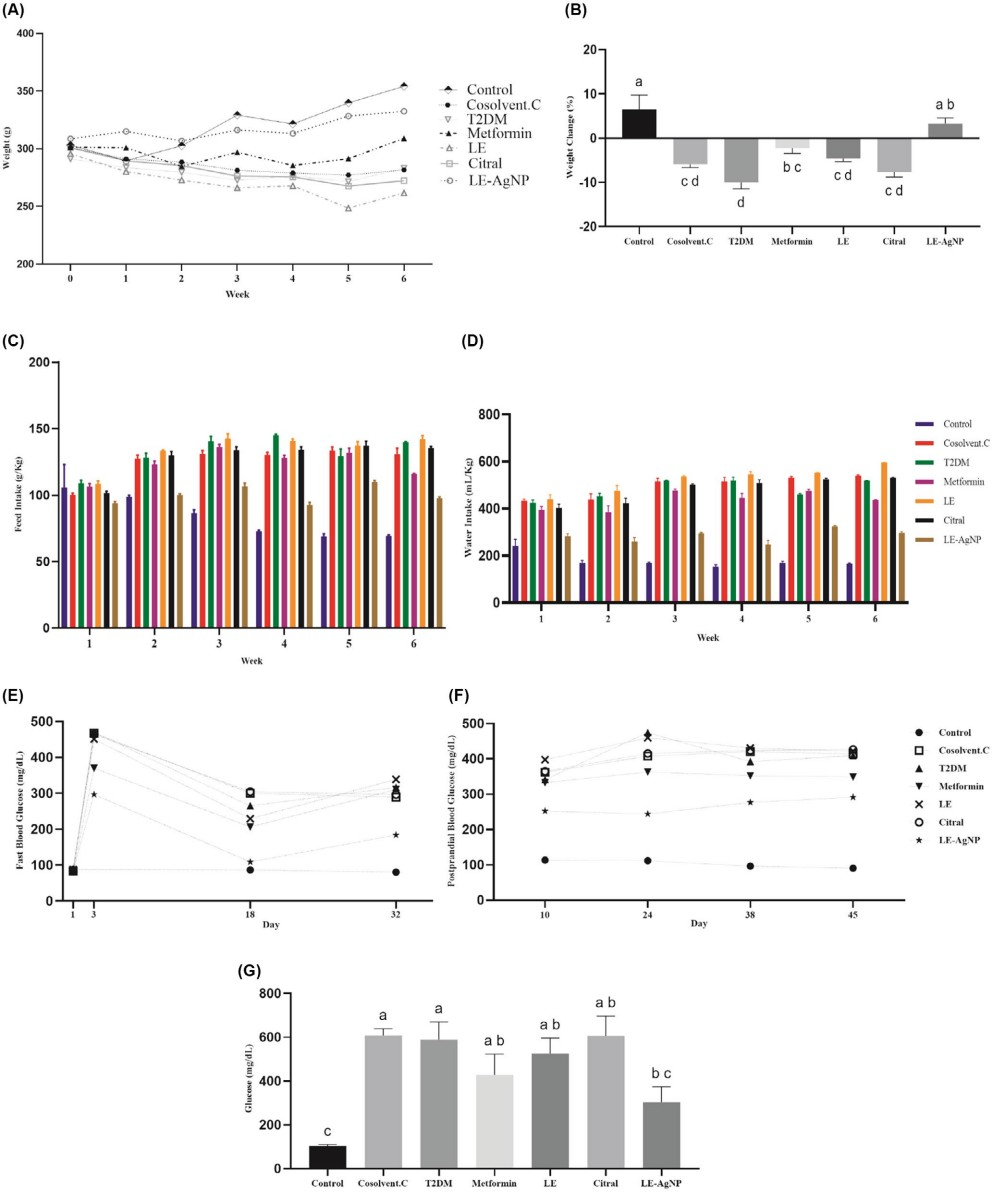
The effects of metformin, LE, citral and LE‐AgNP. (A) Trend of weight changes over time; (B) Percentage weight changes during the research period. (C) Changes in the amount of food consumed. (D) Changes in the amount of water consumed. (E) Trend of fast blood glucose. (F) Trend of postprandial blood glucose. (G) Blood glucose levels at the end of the study. Values are expressed as mean ± SEM. Mean values followed by different superscript letters indicate significant statistical difference (*p* < 0.05). Citral, (T2DM + citral); Cosolvent C, Cosolvent Control (T2DM + citral carrier); LE, Lemongrass extract (T2DM + LE); LE‐AgNP, Silver nanoparticles synthesis by lemongrass extract (T2DM + LE‐AgNP); Metformin, (T2DM + Metformin); T2DM, Type 2 diabetes mellitus.

The control and LE‐AgNP groups showed a positive weight trend, while the other groups had a negative trend (Figure 2A). The percentage of weight change was negative in all diabetic groups studied, except for the group receiving silver nanoparticles of lemongrass extract (Figure 2B).

A significant increase in food consumption was observed among all treatment groups compared to the control (*p* < 0.05), except the LE‐AgNP group (Figure 2C). Water consumption was significantly higher in all groups compared to the control (*p* < 0.05), except in the LE‐AgNP‐treated group, which showed no significant change (*p* > 0.05) (Figure 2D).

Alterations in both fasting (Figure 2E) and postprandial (Figure 2F) blood glucose levels were monitored throughout the experiment. By the third day after STZ and NA injections, fasting glucose levels increased significantly in all groups except the control, confirming successful diabetes induction. Metformin and LE‐AgNP treatment reduced glucose levels compared to the T2DM group, with LE‐AgNP showing a the most reduction. LE and citral did not significantly affect glucose levels (*p* > 0.05). At the end of the experiment, serum blood glucose levels in diabetic groups were significantly higher than in the control group (*p* < 0.0001), but the LE‐AgNP‐treated group showed a significant reduction (Figure 2G).

### 3.3 Changes in TAC and MDA levels

Table 1 represents the value of MDA and TAC in the experimental groups of rats. The TAC levels in both serum and pancreas were reduced in the T2DM and cosolvent control groups compared to other groups. In the treated diabetic groups, TAC levels increased relative to the healthy control group. Specifically, the LE‐AgNP‐treated group exhibited a significant increase in TAC levels compared to the T2DM group in serum (*p* = 0.0487) and pancreas tissue (*p* = 0.0003).

**TABLE 1:** The values of MDA and TAC in the serum and pancreatic tissue of experimental groups of rats.

Groups	MDA		TAC	
	Tissue (μmol/100 mg)	Serum (μmol/100 mL)	Tissue (mmol/100 mg)	Serum (mmol/100 mL)
Control	4.78 ± 0.50^a^	15.40 ± 0.8^d^	0.21 ± 0.02^ab^	0.25 ± 0.01^ab^
Cosolvent. C	7.92 ± 1.38^ab^	44.70 ± 7.41^ab^	0.20 ± 0.01^ab^	0.24 ± 0.01^ab^
T2DM	8.96 ± 0.63^b^	49.68 ± 1.9^a^	0.12 ± 0.01^b^	0.23 ± 0.03^b^
Metformin	6.21 ± 0.70^ab^	26.10 ± 2.7^bd^	0.26 ± 0.01^a^	0.31 ± 0.01^ab^
LE	6.48 ± 0.23^ab^	33.35 ± 7.14^ad^	0.22 ± 0.01^a^	0.30 ± 0.02^ab^
Citral	6.74 ± 0.93^ab^	37.77 ± 3.8^abc^	0.22 ± 0.02^a^	0.26 ± 0.02^ab^
LE‐AgNP	5.78 ± 0.68^ab^	18.40 ± 1.4^cd^	0.27 ± 0.03^a^	0.35 ± 0.03^a^

*Note*: Values are expressed as mean ± SEM. Mean values followed by different superscript letters indicate significant statistical difference (*p* < 0.05).

Abbreviations: Citral, (T2DM + citral); Cosolvent C, Cosolvent Control (T2DM + citral carrier); LE, lemongrass extract (T2DM + LE); LE‐AgNP, silver nanoparticles synthesis by lemongrass extract (T2DM + LE‐AgNP); Metformin, (T2DM + Metformin); T2DM, type 2 diabetes mellitus.

The serum MDA levels significantly increased in the T2DM (*p* = 0.0001) and cosolvent control groups (*p* = 0.0019) compared to the healthy control group. Although the treated groups showed elevated MDA levels, these increases were not statistically significant (*p* > 0.05). However, treatment with LE‐AgNP (*p* = 0.0005) and metformin (*p* = 0.0124) effectively mitigated the rise in MDA levels compared to the T2DM group. Similarly, tissue MDA levels were significantly higher in the T2DM group compared to the healthy control group (*p* = 0.0398).

### 3.4 Changes in total protein, Nrf2 gene expression and Nrf2 protein level

**FIGURE 3:**
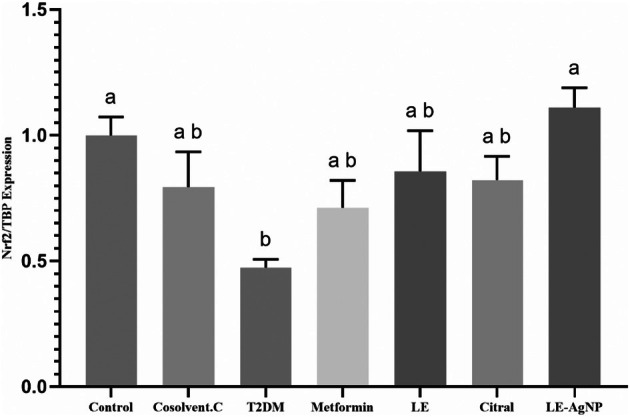
The effects of metformin, LE, citral and LE‐AgNP on Nrf2 gene expression level in pancreas tissue. Values are expressed as mean ± SEM. Mean values followed by different superscript letters indicate significant statistical difference (*p* < 0.05). Citral, (T2DM + citral); Cosolvent C, Cosolvent Control (T2DM + citral carrier); LE, Lemongrass extract (T2DM + LE); LE‐AgNP, Silver nanoparticles synthesis by lemongrass extract (T2DM + LE‐AgNP), TBP, TATA Binding Protein; Metformin, (T2DM + Metformin); T2DM, Type 2 diabetes mellitus.

At the end of study, Nrf2 gene expression in pancreatic tissue decreased across all groups except for the LE‐AgNP group. The reduction in gene expression was statistically significant in the T2DM group compared to the healthy control group (*p* = 0.019) (Figure 3). Oral administration of metformin, LE, citral, and LE‐AgNP over 6 weeks did not significantly affect Nrf2 protein levels in serum and pancreatic tissue compared to control groups. Additionally, total protein levels in pancreatic tissue showed no significant differences between the groups. Serum total protein levels were generally consistent across groups, with a significant increase observed only in the metformin‐treated group compared to the cosolvent control and T2DM groups (Table 2).

**TABLE 2:** The values of NRF2 in the serum and pancreatic tissue of experimental groups of rats.

Groups	Nrf2		Total Protein	
	Tissue (ng/mg tissue)	Serum (mmol/100 mg)	Tissue (μg/mL)	Serum (g/dL)
Control	3.30 ± 0.32^a^	2.03 ± 0.14^a^	10.09 ± 1.52^a^	6.56 ± 0.28^ab^
Cosolvent C	2.66 ± 0.33^a^	1.58 ± 0.16^a^	9.83 ± 0.76^a^	6.36 ± 0.11^b^
T2DM	2.13 ± 0.50^a^	1.31 ± 0.09^a^	9.40 ± 1.40^a^	6.34 ± 0.15^b^
Metformin	2.61 ± 0.36^a^	1.57 ± 0.18^a^	12.78 ± 1.01^a^	7.54 ± 0.43^a^
LE	2.91 ± 0.27^a^	1.73 ± 0.11^a^	10.84 ± 0.53^a^	6.42 ± 0.34^ab^
Citral	2.85 ± 0.20^a^	1.79 ± 0.24^a^	10.73 ± 0.87^a^	6.66 ± 0.14^ab^
LE‐AgNP	3.41 ± 0.47^a^	1.96 ± 0.13^a^	14.09 ± 1.37^a^	6.84 ± 0.13^ab^

*Note*: Values are expressed as mean ± SEM. Mean values followed by different superscript letters indicate significant statistical difference (*p* < 0.05).

Abbreviations: Citral, (T2DM + citral); Cosolvent C, Cosolvent Control (T2DM + citral carrier); LE, lemongrass extract (T2DM + LE); LE‐AgNP, silver nanoparticles synthesis by lemongrass extract (T2DM + LE‐AgNP); Metformin, (T2DM + Metformin); T2DM, type 2 diabetes mellitus.

### 3.5 Histopathology and morphometry changes

**FIGURE 4:**
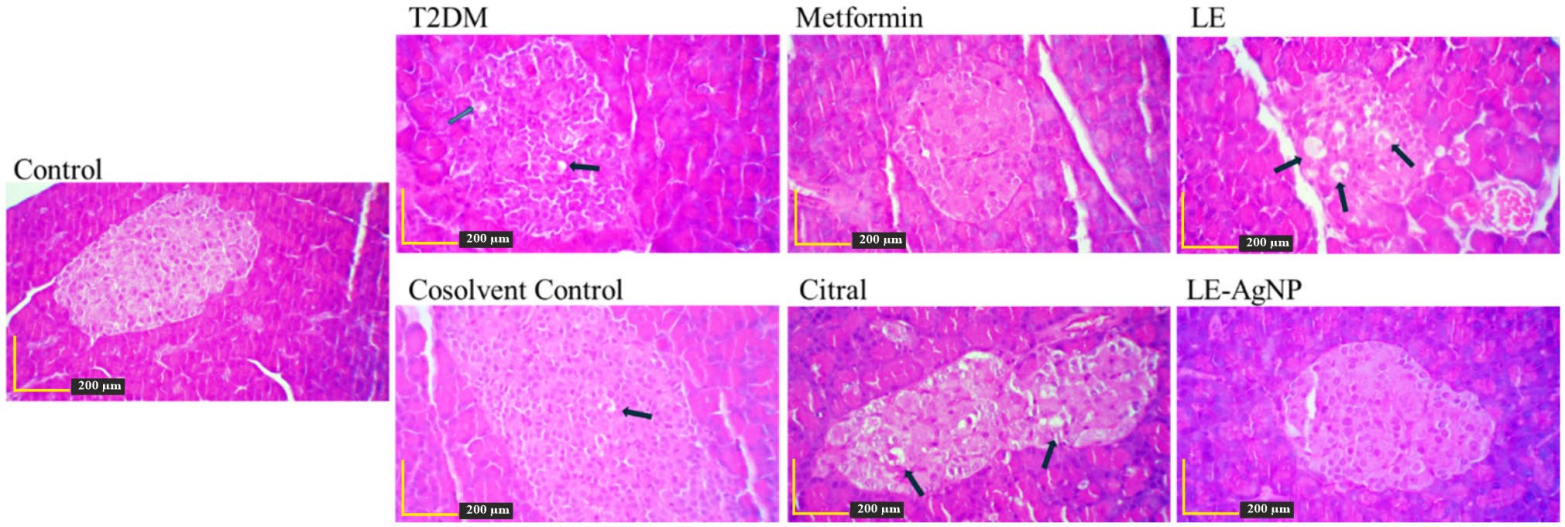
The histostructure of the pancreatic tissue by Hematoxylin–Eosin staining. Magnification: ×400. Citral, (T2DM + citral); Cosolvent C, Cosolvent Control (T2DM + citral carrier); LE, Lemongrass extract (T2DM + LE); LE‐AgNP, Silver nanoparticles synthesis by lemongrass extract (T2DM + LE‐AgNP); Metformin, (T2DM + Metformin); T2DM, Type 2 diabetes mellitus.

The histostructure and histopathology of the pancreatic tissue of the 7 studied groups is shown by Hematoxylin–Eosin staining in Figure 4. In the control group, the pancreatic islets and the alpha and beta cells were intact. The T2DM group showed signs of hypertrophy and vacuolation. Similar vacuolation was observed in the groups treated with LE and citral. However, in the LE‐AgNP and metformin‐treated groups, the islet structure showed notable improvements, with no observed complications.

**FIGURE 5:**
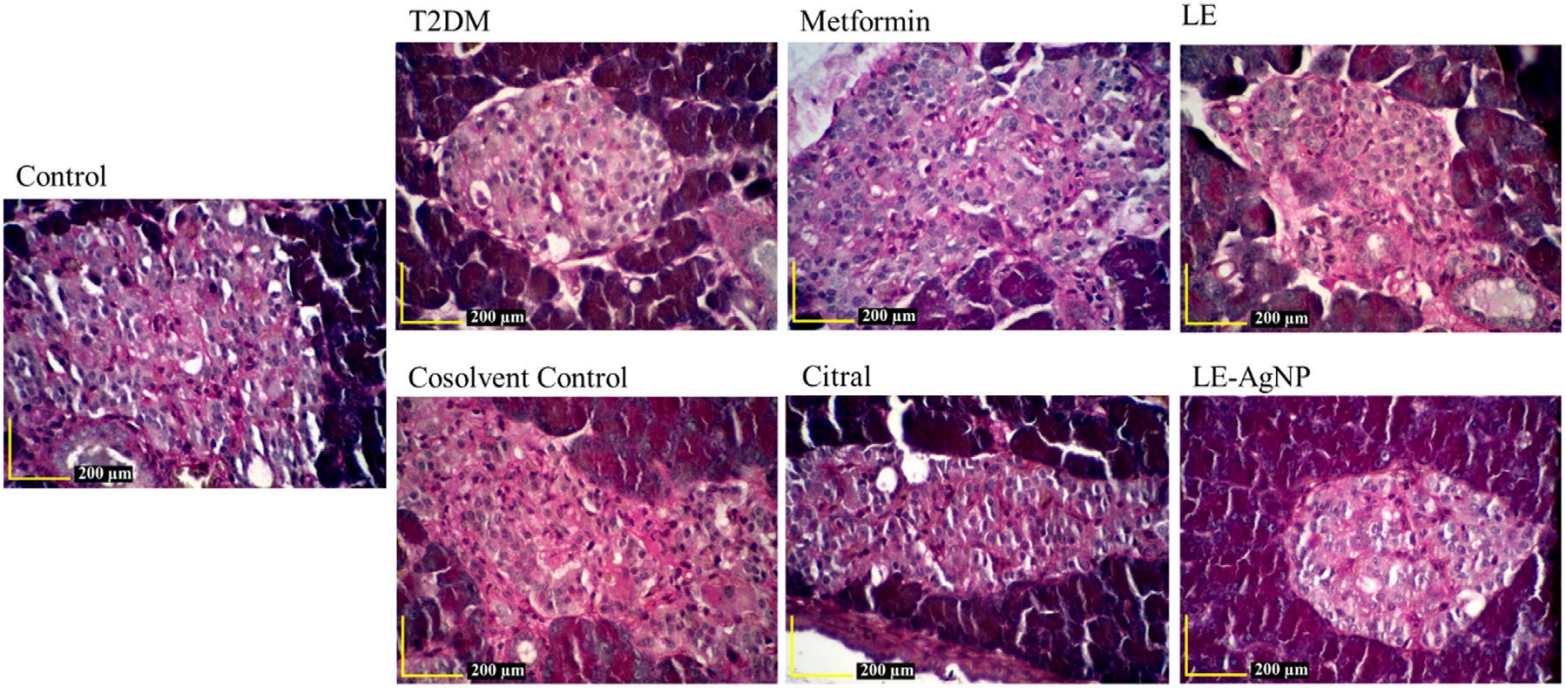
The histostructure of the pancreatic tissue by Gomori staining. Magnification ×400. Citral, (T2DM + citral); Cosolvent C, Cosolvent Control (T2DM + citral carrier); LE, Lemongrass extract (T2DM + LE); LE‐AgNP, Silver nanoparticles synthesis by lemongrass extract (T2DM + LE‐AgNP); Metformin, (T2DM + Metformin); T2DM, Type 2 diabetes mellitus.

**FIGURE 6:**
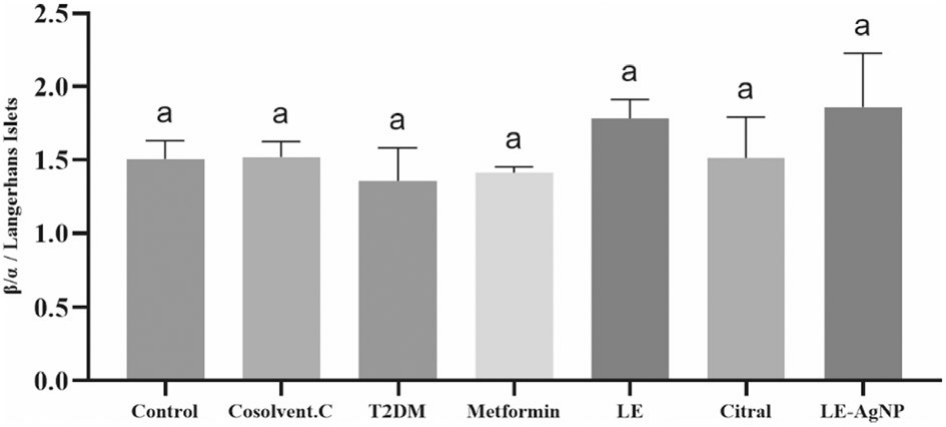
The effects of metformin, LE, citral and LE‐AgNP on ratio of beta to alpha cells in the islets of Langerhans levels. Values are expressed as mean ± SEM. Mean values followed by different superscript letters indicate significant statistical difference (*p* < 0.05). Citral, (T2DM + citral); Cosolvent C, Cosolvent Control (T2DM + citral carrier); LE, Lemongrass extract (T2DM + LE); LE‐AgNP, Silver nanoparticles synthesis by lemongrass extract (T2DM + LE‐AgNP); Metformin, (T2DM + Metformin); T2DM, Type 2 diabetes mellitus.

Gomori staining differentiated the islets, marking alpha cells as red and beta cells as blue (Figure 5). The micrometric analysis of Langerhans islets showed no significant changes in the beta‐to‐alpha cell ratio among the studied groups. However, a decrease in this ratio was observed in the T2DM group compared to the control, indicating successful diabetes induction in the rats (Figure 6).

## 4 DISCUSSION

Streptozotocin is a derivative of 2‐deoxy‐D‐glucose, featuring an N‐nitrosomethylurea moiety in its structure, readily absorbed by Glut‐2 transporters in pancreatic beta cells. Inside these cells, it generates free radicals, causing DNA fragmentation and necrosis, ultimately reducing insulin synthesis.^26^ Administering NA before STZ preserves 40% of insulin reserves in pancreatic cells. This model induces persistent moderate hyperglycemia, glucose intolerance, overeating, weight loss, and other diabetes symptoms.^22^ Therefore, STZ and NA‐induced diabetes in rats was chosen for this research.

During the six‐week experiment, all treated groups showed significantly increased food and water consumption compared to the control group. It is important to note that the group treated with nanoparticles exhibited a comparatively smaller increase. This aligns with previous findings on water consumption.^27^, ^28^, ^29^ However, treated diabetic groups consumed more food than the control group, contradicting earlier studies. Weight changes were inversely proportional to food and water intake; diabetic groups experienced weight loss despite increased consumption, consistent with diabetic conditions and past research. Conversely, the diabetic group treated with nanoparticles gained weight, indicating silver nanoparticles may improve diabetic conditions.

Citral did not reduce blood glucose levels in comparison to the T2DM group, contrary to previous finding.^29^ However, metformin, LE, and LE‐AgNP lowered blood glucose levels, as observed in previous study.^28^ Notably, only LE‐AgNP significantly reduced blood glucose after six weeks, likely due to the hypoglycemic properties of silver nanoparticles.^30^

The diabetes control groups showed a non‐significant reduction in total serum protein compared to the control, possibly due to liver damage, absorption disorders, and nephropathy, which contrasts with previous studies.^28^ Treatment groups showed no significant differences, but metformin increased total protein, aligning with prior research.^28^, ^31^ Pancreatic total protein levels followed similar trends, with no significant differences between groups. However, the group treated with silver nanoparticles showed a higher increase in pancreatic protein, likely due to nanoparticle‐enhanced cell permeability, potentially improving the synthesis of pancreatic proteins like pro‐insulin, insulin, and exogenous enzymes. This finding warrants further examination of pancreatic synthetic protein levels.

The results showed that TAC levels were reduced in the T2DM and cosolvent control groups compared to the healthy control, likely due to oxidative stress caused by hyperglycemia. In contrast, the diabetic treatment groups had higher tissue and serum TAC levels than the healthy group, aligning with previous studies.^31^, ^32^ Notably, the LE‐AgNP group exhibited significantly higher TAC levels in both serum and tissue compared to the T2DM group.

Diabetes induces oxidative stress by elevating hyperglycemia and hyperlipidemia, leading to lipid peroxidation, which can be quantified by MDA concentrations. Increased MDA levels indicate higher lipid peroxidation.^33^ In the present investigation, pancreatic tissue MDA concentrations were elevated in all diabetic groups compared to the healthy group, consistent with earlier studies.^14^, ^32^, ^34^ The T2DM group showed the most significant increase. However, the LE‐AgNP treated diabetic group exhibited the smallest rise in MDA levels, likely due to LE‐Ag‐NP's hypoglycemic properties reducing free radical generation and lipid peroxidation. Serum MDA levels mirrored those in pancreatic tissue, with all diabetic groups showing increased levels compared to the healthy control group, especially the T2DM group. Notably, the cosolvent control group showed a greater increase in serum MDA than in pancreatic tissue, possibly due to the lipid‐rich nature of the citral carrier enhancing lipid peroxidation, which contrasts with previous studies.^35^, ^36^

Nrf2 is a key regulator of the antioxidant response.^37^ Under normal conditions, Nrf2 remains inactive inside cells due to its association with Keap1 (Kelch‐like ECH‐associated protein 1). However, reactive oxygen species disrupt this complex, releasing Nrf2, which is then activated through protein kinase B (AKT) activity and subsequent phosphorylation.^38^, ^39^ Activated Nrf2 binds to Antioxidant Response Element (ARE) regions in the promoter of genes involved in cellular defense, enhancing the cell's response to oxidative and electrophilic stressors,^40^, ^41^ and also regulates its own mRNA transcription.^39^

Protein phosphatase 2 (PP2A) is a serine/threonine phosphatase that dephosphorylates and inhibits AKT and its downstream signaling pathways.^42^ Studies suggest that ROS can inhibit PP2A by oxidizing cysteine residues, reducing its ability to deactivate AKT. In environments with low ROS levels, oxidative conditions can lead to the formation of disulfide (‐S‐S‐) bonds between cysteine residues in AKT, enhancing the interaction between AKT and PP2A, which in turn inhibits AKT activity and its downstream pathways, including Nrf2.^43^, ^44^ This regulation supports the activation of cellular defense mechanisms against oxidative stress, helping to maintain cellular balance and protecting against oxidative damage‐related pathologies.

The analysis of Nrf2 gene expression in pancreatic tissue revealed significant differences across diabetic groups. In the T2DM group, Nrf2 gene expression was significantly lower compared to the healthy control group. This reduction may be attributed to elevated levels of ROS, which inactivate AKT, leading to decreased activation and expression of the Nrf2 gene. In contrast, diabetic groups treated with metformin, citral, and LE showed a non‐significant decrease in Nrf2 expression compared to the healthy group, suggesting these compounds help maintain basal Nrf2 levels. Notably, the diabetic group treated with LE‐AgNP showed a significant increase in Nrf2 expression compared to the T2DM group, indicating its strong therapeutic and protective effects. This increase suggests that LE‐AgNP not only mitigate ROS—which suppress Nrf2 expression—but also enhance Nrf2 gene expression, with levels even surpassing those in the healthy control group. These findings align with previous studies showing that prolonged exposure to fluctuating glucose levels in T2DM reduces Nrf2 expression.^45^, ^46^, ^47^, ^48^, ^49^ The study's findings demonstrate that treatments with metformin, LE, and citral effectively prevented a substantial reduction in Nrf2 gene expression, as illustrated in Figure 3. In the diabetic group treated with Ag‐NP, Nrf2 expression significantly increased, indicating that these treatments may work by inducing Nrf2 expression in pancreatic tissue. This induction is likely due to their ability to reduce low levels of ROS, which are common in type 2 diabetes and act as suppressors of Nrf2 gene expression and protein synthesis. As a result, Nrf2 levels are restored to their normal state within cells. Among the tested treatments, LE‐AgNP proved to be the most effective. Their superior efficacy can be attributed to improvements in blood glucose regulation, enhanced antioxidant levels, greater stability, and improved cellular penetration, making them a more potent therapeutic option for managing oxidative stress and supporting Nrf2 expression in diabetes. Furthermore, analysis of Nrf2 protein levels in serum and tissue using ELISA kits showed no significant changes; however, the Nrf2 protein levels in pancreatic tissue corresponded with the observed gene expression patterns, confirming the results of the real‐time PCR analysis.

## 5 CONCLUSION

In conclusion, the oral administration of LE, citral, LE‐AgNP, and metformin demonstrated antioxidant effects by improving Nrf2 levels and reducing oxidative stress. Metformin effectively lowered blood glucose, while LE and citral showed moderate protective effects. LE‐AgNP exhibited the most potent results, significantly enhancing Nrf2 expression, regulating blood glucose, stabilizing food and water intake, preventing weight loss, and reducing pancreatic histopathological changes. These findings highlight the therapeutic potential of LE‐AgNP in managing oxidative stress and improving diabetic conditions.

## AUTHOR CONTRIBUTIONS

**Milad Faraji:** Conceptualization; formal analysis; methodology; writing – original draft. **Mohammad Foad Noorbakhsh:** Investigation; resources; validation; writing – review and editing. **Nasrin Kazemipour:** Investigation; resources; supervision; writing – review and editing. **Saeed Nazifi:** Methodology; resources. **Hamid Reza Moradi:** Methodology; validation. **Nasrollah Ahmadi:** Methodology; validation. **Maryam Azadmanesh:** Methodology.

## FUNDING INFORMATION

All the experiments expenses supported by the Shiraz University, Shiraz, Iran.

## CONFLICT OF INTEREST STATEMENT

All authors declare that they have no conflicts of interest.

## ETHICS STATEMENT

All experimental procedures involving laboratory animals were conducted in accordance with the biological ethics regulations issued by Shiraz University's Research Vice‐Chancellor (No. 1GCB1M348200).
